# Further Study of Multigranulation *T*-Fuzzy Rough Sets

**DOI:** 10.1155/2014/927014

**Published:** 2014-08-17

**Authors:** Wentao Li, Xiaoyan Zhang, Wenxin Sun

**Affiliations:** School of Mathematics and Statistics, Chongqing University of Technology, Chongqing 400054, China

## Abstract

The optimistic multigranulation *T*-fuzzy rough set model was established based on multiple granulations under *T*-fuzzy approximation space by Xu et al., 2012. From the reference, a natural idea is to consider pessimistic multigranulation model in *T*-fuzzy approximation space. So, in this paper, the main objective is to make further studies according to Xu et al., 2012. The optimistic multigranulation *T*-fuzzy rough set model is improved deeply by investigating some further properties. And a complete multigranulation *T*-fuzzy rough set model is constituted by addressing the pessimistic multigranulation *T*-fuzzy rough set. The full important properties of multigranulation *T*-fuzzy lower and upper approximation operators are also presented. Moreover, relationships between multigranulation and classical *T*-fuzzy rough sets have been studied carefully. From the relationships, we can find that the *T*-fuzzy rough set model is a special instance of the two new types of models. In order to interpret and illustrate optimistic and pessimistic multigranulation *T*-fuzzy rough set models, a case is considered, which is helpful for applying these theories to practical issues.

## 1. Introduction

Rough set theory, proposed by Pawlak [1], is an extension of the classical set theory and could be regarded as a mathematical and soft computing tool to handle imprecision, vagueness, and uncertainty in data analysis. This relatively new soft computing methodology has received great attention in recent years, and its effectiveness has been confirmed successful in applications in many science and engineering fields, such as pattern recognition, data mining, image processing, and medical diagnosis. Rough set theory is built on the basis of the classification mechanism; it is classified as the equivalence relation in a specific universe, and the equivalence relation constitutes a partition of the universe. A concept, or more precisely the extension of a concept, is represented by a subset of a universe of objects and is approximated by a pair of definable concepts of a logic language. The main idea of rough set theory is the use of a known knowledge in knowledge base to approximate the inaccurate and uncertain knowledge.

In recent years, the generalization of the rough set model is one of the most important research directions. On one hand, rough set theory is generalized by combining with other theories that deal with uncertain knowledge such as fuzzy set theory [2]. It has been acknowledged by different studies that fuzzy set theory and rough set theory are complementary in terms of handling different kinds of uncertainty. Fuzzy set theory deals with probabilistic uncertainty, connected with imprecision of stated perceptions and preferences. Rough set theory, in turn, deals with uncertainty following from ambiguity of information. The two types of uncertainty can be encountered together in real-life problems. For this reason, many approaches have been proposed to combine fuzzy set theory with rough set theory. D$\ddot{\text{u}}$bois and Prade [3] proposed concepts of rough fuzzy sets and fuzzy rough sets. A rough fuzzy set is a pair of fuzzy sets resulting from the approximation of a crisp set in a fuzzy approximation space, and a fuzzy rough set is a pair of fuzzy sets resulting from the approximation of a fuzzy set in a crisp approximation space. Some other researches about fuzzy rough set and rough fuzzy set from other directions have been discussed [4–10]. What is more, generalizations of fuzzy rough sets were defined by using a residual implication and a triangular norm on [0,1] to define the lower and upper approximation operators. Several authors also have proposed a kind of implication 4, weak fuzzy partitions on the universe. Wu et al. [11] characterized the (*I*, *T*)-fuzzy rough approximation operators. Morsi and Yakout researched axiomatics for fuzzy rough sets by a triangular norm [12]. Mi et al. generalized fuzzy rough sets determined by a triangular norm [13].

On the other hand, rough set theory was discussed from the view of granular computing. In 1985, Hobbs proposed the concept of granularity [14], and Zadeh first explored the concept of granular computing between 1996 and 1997 [15]. They all think that information granules refer to pieces, classes, and groups into which complex information is divided in accordance with the characteristics and processes of the understanding and decision-making. Among the existing possibilities offered by granular computing, we may refer to fuzzy sets [16], rough sets [1], and vague sets [17], just to name some of the well-established alternatives. From the point of view of granular computing, Pawlak's rough set is based on a single granulation induced from an indiscernibility relation.

Actually, an attribute subset induces an equivalence relation; the partition formed by an equivalence relation can be regarded as a granulation. By using a finer granulation formed through combining two known granulations induced from two-attribute subsets to describe the target concept, the combination destroys the original granulation structure. In general, the above assumption cannot always be satisfied or required in practice. In order to apply the rough set theory, Qian and Liang extended Pawlak's single-granulation rough set model to a multiple granulation rough set model [18]. Since the multigranulation rough set was initially proposed by Qian et al. [19], later, many researchers have extended the multigranulation rough sets to the generalized multigranulation rough sets. Xu et al. developed a multigranulation fuzzy rough set model [20], a generalized multigranulation rough set approach [21], multigranulation rough sets based on tolerance relations [22], and a multigranulation rough set model in ordered information systems [23]; Yang et al. proposed the hierarchical structure properties of the multigranulation rough sets [24] and multigranulation rough set in incomplete information system [25] and presented a test cost sensitive multigranulation rough set model [26]; Lin et al. presented a neighborhood-based multigranulation rough set [27]; She and He explored the topological structures and the properties of multigranulation rough sets [28].

From the thought of multigranulation, optimistic multigranulation and pessimistic multigranulation are two of the most basic ways of research. In [29], authors only presented concepts of optimistic multigranulation fuzzy rough sets based on triangular norms. By analyzing the proposed definition in [29], there exists another perspective which is called pessimistic multigranulation. Authors in [29] did not investigate the pessimistic multigranulation fuzzy rough sets based on triangular norms, and relationships between optimistic multigranulation and single granulation fuzzy rough sets based on triangular norms were not presented either. Accordingly, from both optimistic multigranulation and pessimistic multigranulation perspectives, we generalize the multigranulation *T*-fuzzy rough set theory by using the concepts of a residual implication and a triangular norm on [0,1]. In this paper, we mainly improve the model proposed in [29] by discussing the further properties of optimistic multigranulation *T*-fuzzy rough sets, propose the multigranulation *T*-fuzzy rough set model from the perspective of pessimistic multigranulation and study its properties, and research relationships between multigranulation and classical *T*-fuzzy rough sets. These contents are not yet completed in [29], so this paper is an extended vision of [29]. The rest of this paper is organized as follows. In Section 2, we recall some concepts and properties to be used in this paper. In Sections 3 and 4, we presented the definition of the optimistic multigranulation *T*-fuzzy lower and upper approximation operators and proposed the pessimistic multigranulation *T*-fuzzy lower and upper approximation operators; basic properties about these two models are also studied. In Section 5, we get the relationship among these *T*-fuzzy approximation operators. We give the examples about the evaluation of fund projects in Section 6. Finally, Section 7 gets the conclusions.

## 2. Preliminaries

In this section, we review some basic concepts and properties about *T*-fuzzy rough sets. The notion of optimistic multigranulation *T*-fuzzy rough set is also introduced. The Cartesian product of *U* with *U* is denoted by *U* × *U*. The classes of all fuzzy subsets of *U* are denoted by *F*(*U*). Following, a binary operator *T* on the unit interval *I* = [0,1] is said to be a triangular norm [30] if for all *a*, *b*, *c*, *d* ∈ *I*, we have

- *T*(*a*, *b*) = *T*(*b*, *a*);
- *T*(*a*, 1) = *a*;
- *a* ≤ *c*, *b* ≤ *d*⇒*T*(*a*, *b*) ≤ *T*(*c*, *d*);
- *T*(*T*(*a*, *b*), *c*) = *T*(*a*, *T*(*b*, *c*)).

A fuzzy relation *R* from *U* to *U* is a fuzzy subset of *U* × *U*; that is, *R* ∈ *F*(*U* × *U*), and *R*(*x*, *y*) is called the degree of relation between *x* and *y*. Consider the following:

1. *R* is said to be reflexive on *U* × *U*⇔ for all *x* ∈ *U*, *R*(*x*, *x*) = 1;
2. *R* is said to be symmetric on *U* × *U*⇔ for all *x* ∈ *U*, *R*(*x*, *y*) = *R*(*y*, *x*);
3. *R* is said to be *T* transitive on *U* × *U*⇔ for all *x*, *y*, *z* ∈ *U*, *R*(*x*, *z*) ≥ *T*(*R*(*x*, *y*), *R*(*y*, *z*)).

If *R* is reflexive, symmetric, and *T* transitive on *U* × *U*, we then say that *R* is a *T*-fuzzy equivalence relation on *U*; if *R* is reflexive and symmetric on *U* × *U*, we say that *R* is a *T*-fuzzy similarity relation on *U*.

A binary operator on *I* is given in the following

(1) $$\begin{array}{c} \theta \left(a,b\right)=\sup\left\{c\in I\;|\;T\left(a,c\right)\leq b\right\}, \end{array}$$

where *θ*  is called the residual implication based on a triangular norm *T*.

For the sake of convenience, for any *X*, *Y* ∈ *F*(*U*), *x* ∈ *U*, we will define several fuzzy sets as follows:

(1) *T*(*X*, *Y*)(*x*) = *T*(*X*(*x*), *Y*(*x*));
(2) *θ*(*X*, *Y*)(*x*) = *θ*(*X*(*x*), *Y*(*x*));
(3) *θ*(*X*, *Y*) = ⋀_*u*∈*U*_ *θ*(*X*(*u*), *Y*(*u*));
(4) $\theta (1_{x},\underline{\underline{a}})=1_{U\setminus \{x\}}\vee \underline{\underline{a}}$;
(5) (2) $$\begin{array}{c} 1_{x}\left(y\right)=\{\begin{array}{cc} 1, & \text{if}\;\;y=x;\\ 0, & y\neq x; \end{array} \end{array}$$
(6) (3) $$\begin{array}{c} 1_{Z}\left(x\right)=\{\begin{array}{cc} 1, & \text{if}\;\;x\in Z;\\ 0, & x\notin Z, \end{array} \end{array}$$

where $\underset{=}{a}$ is a constant fuzzy set and *Z*⊆*U*.

Consider a lower semicontinuous triangular norm *T*, for all *a*, *b*, *c* ∈ *I*; the residual implication based on the triangular norm *T* satisfies the following important properties:

(*θ*1)
*θ*(*a*, 1) = 1, *θ*(1, *a*) = *a*;
(*θ*2)
*a* ≤ *b*⇒*θ*(*c*, *a*) ≤ *θ*(*c*, *b*);
(*θ*3)
*a* ≤ *b*⇒*θ*(*a*, *c*) ≥ *θ*(*b*, *c*);
(*θ*4)
*T*(*θ*(*a*, *c*), *θ*(*c*, *b*)) ≤ *θ*(*a*, *b*);
(*θ*5)
*θ*(*a*∨*b*, *c*) = *θ*(*a*, *c*)∧*θ*(*b*, *c*);
(*θ*6)
*θ*(*a*, *b*∧*c*) = *θ*(*a*, *b*)∧*θ*(*a*, *c*);
(*θ*7)
*a* ≤ *b*⇔*θ*(*a*, *b*) = 1;
(*θ*8)
*θ*(*a*, *θ*(*b*, *c*)) = *θ*(*b*, *θ*(*a*, *c*));
(*θ*9)
*θ*(*T*(*a*, *b*), *c*) = *θ*(*a*, *θ*(*b*, *c*));
(*θ*10)
*T*(*θ*(*T*(*a*, *b*), *c*), *a*) ≤ *θ*(*b*, *c*);
(*θ*11)
⋀_*a*∈*I*_ *θ*(*T*(*b*, *θ*(*c*, *a*)), *a*) = *θ*(*b*, *c*);
(*θ*12)
*θ*(*θ*(*a*, *b*), *b*) ≥ *a*;
(*θ*13)
⋀_*b*∈*I*_ *θ*(*θ*(*a*, *b*), *b*) = *a*;
(*θ*14)
*T*(*θ*(*a*, *b*), *c*) ≤ *θ*(*a*, *T*(*b*, *c*));
(*θ*15)
⋀_*b*∈*I*_ *θ*(*θ*(*a*, *b*), *θ*(*c*, *b*)) = *θ*(*c*, *a*);
(*θ*16)
*θ*(*a*, *b*) ≤ *θ*(*T*(*a*, *c*), *T*(*b*, *c*));
(*θ*17)
*θ*(*a*, *b*∨*c*) = *θ*(*a*, *b*)∨*θ*(*a*, *c*);
(*θ*18)
*a* ≤ *θ*(*b*, *T*(*a*, *b*));
(*θ*19)
*θ*(*a*∧*b*, *c*) = *θ*(*a*, *c*)∨*θ*(*b*, *c*);
(*θ*20)
*θ*(*a*∧*b*, *c*) ≥ *θ*(*a*, *c*)∧*θ*(*b*, *c*).

**Definition 1 (see [13]).**

Let *U* be a finite and nonempty set called the universe, and let *R* be a *T*-fuzzy similarity relation from *U* to *U*. The pair (*U*, *R*) is called a *T*-fuzzy approximation space. For any *A* ∈ *F*(*U*), we define two fuzzy set-theoretic operators from *F*(*U*) to *F*(*U*) as follows:

(4) R_(A)(x)=⋀y∈Uθ(R(x,y),A(y)),R¯(A)(x)=⋁y∈UT(R(x,y),A(y)),x∈U,

where “⋁” means “max,” “⋀” means “min,” and $\underline{R}$ and $\bar{R}$ are referred to as the *T*-fuzzy lower and upper approximation operators. The pair $\left(\underline{R}(A),\bar{R}(A)\right)$ is called the *T*-fuzzy rough set of *A*.

**Proposition 2 (see [13]).**

Let (*U*, *R*) be a fuzzy approximation space, for all *A*, *B* ∈ *F*(*U*), (*x*, *y*) ∈ *U* × *U*; then, one has the following:

1. $\underline{R}(A)\subseteq A\subseteq \bar{R}(A)$;
2. $\underline{R}(A\cap B)=\underline{R}(A)\cap \underline{R}(B)$, $\bar{R}((A)\cup B)=\bar{R}(A)\cup \bar{R}(B)$;
3. $\underline{R}(A\cup B)⊇\underline{R}(A)\cup \underline{R}(B)$, $\underline{R}(A\cap B)\subseteq \underline{R}(A)\cap \bar{R}(B)$;
4. $A\subseteq B\Rightarrow \underline{R}(A)\subseteq \underline{R}(B)$, $\bar{R}(A)\subseteq \bar{R}(B)$;
5. $\underline{R}(\underline{R}(A))=\underline{R}(A)$, $\bar{R}(\bar{R}(A))=\bar{R}(A)$;
6. $\bar{R}(\underline{R}(A))=\underline{R}(A)$, $\underline{R}(\bar{R}(A))=\bar{R}(A)$;
7. $\bar{R}(A)=A\Leftrightarrow \underline{R}(A)=A$.

The above proposition reflects the relationships between $\underline{R}$ and $\bar{R}$. It is easy to prove that the *T*-fuzzy approximation operators in this section are really extensions of the approximation operators. In particular, if *R* is a crisp equivalence relation on *U* and *A* ∈ *P*(*U*), then $\bar{R}(A)$ and $\underline{R}(A)$ coincide with the classical Pawlak rough set.

Authors in [29] proposed the model on optimistic multigranulation *T*-fuzzy rough set, which is presented in the following.

**Definition 3 (see [29]).**

Let *K* = (*U*, **R**) be a *T*-fuzzy approximation space, and let **R** be a family of *T*-fuzzy similarity relations from *U* to *U*, *R*_*A*_1__, *R*_*A*_2__,…, *R*_*A*_*n*__ ∈ **R**. For any *X* ∈ *F*(*U*), the optimistic multigranulation *T*-fuzzy lower and upper approximations of *X* are defined as follows:

(5) $$\begin{array}{c} \underline{{\text{OM}}_{\sum_{i=1}^{n}A_{i}}}\left(X\right)\left(x\right)=\overset{n}{\underset{i=1}{⋁}}\left(\underset{u\in U}{⋀}\theta \left(R_{A_{i}}\left(u,x\right),X\left(u\right)\right)\right),\\ \bar{{\text{OM}}_{\sum_{i=1}^{n}A_{i}}}\left(X\right)\left(x\right)=\overset{n}{\underset{i=1}{⋀}}\left(\underset{u\in U}{⋁}T\left(R_{A_{i}}\left(u,x\right),X\left(u\right)\right)\right), \end{array}$$

where *θ* and *T* are defined in Section 2. $\underline{{\text{OM}}_{\sum_{i=1}^{n}A_{i}}}$ and $\bar{{\text{OM}}_{\sum_{i=1}^{n}A_{i}}}$ are referred to as the optimistic multigranulation *T*-fuzzy lower and upper approximation operators. The pair $\left(\underline{\text{O}{\text{M}}_{\sum_{i=1}^{n}A_{i}}}(X),\bar{\text{O}{\text{M}}_{\sum_{i=1}^{n}A_{i}}}(X)\right)$ is called the optimistic multigranulation *T*-fuzzy rough set of *X*. If $\underline{\text{O}{\text{M}}_{\sum_{i=1}^{n}A_{i}}}(X)=\bar{\text{O}{\text{M}}_{\sum_{i=1}^{n}A_{i}}}(X)$, then *X* is referred to as optimistic definable under the *T*-fuzzy approximation space; otherwise, *X* is referred to as optimistic undefinable or rough. The boundary of the optimistic multigranulation *T*-fuzzy rough set *X* is defined as

(6) $$\begin{array}{c} \text{Bn}{\text{d}}_{\sum_{i=1}^{n}A_{i}}^{O}\left(X\right)=\bar{\text{O}{\text{M}}_{\sum_{i=1}^{n}A_{i}}}\left(X\right)\cap \left(\sim \underline{\text{O}{\text{M}}_{\sum_{i=1}^{n}A_{i}}}\left(X\right)\right). \end{array}$$

**Example 4 (see [29]).**

Let (*U*, *R*_*A*_, *R*_*B*_) be a *T*-fuzzy approximation space, where *U* = {*x*_1_, *x*_2_, *x*_3_, *x*_4_, *x*_5_}; then,

(7) $$\begin{array}{c} R_{A}=\left(\begin{array}{ccccc} 1 & 0.4 & 0.8 & 0.5 & 0.5\\ 0.4 & 1 & 0.4 & 0.4 & 0.4\\ 0.8 & 0.4 & 1 & 0.5 & 0.5\\ 0.5 & 0.4 & 0.5 & 1 & 0.6\\ 0.5 & 0.4 & 0.5 & 0.6 & 1 \end{array}\right),\\ R_{B}=\left(\begin{array}{ccccc} 1 & 0.8 & 0.8 & 0.2 & 0.8\\ 0.8 & 1 & 0.85 & 0.2 & 0.85\\ 0.8 & 0.85 & 1 & 0.2 & 0.9\\ 0.2 & 0.2 & 0.2 & 1 & 0.2\\ 0.8 & 0.85 & 0.9 & 0.2 & 1 \end{array}\right). \end{array}$$

Given *T*(*x*, *y*) = min⁡(*x*, *y*), *X* = (0.5,0.3,0.3,0.6,0.5).

It is not difficult to verify that the fuzzy relations *R*_*A*_ and *R*_*B*_ are both *T*-fuzzy similarity relations. So we can obtain the optimistic multigranulation *T*-fuzzy lower and upper approximations of *X* as follows:

(8) $$\begin{array}{c} \underline{\text{O}{\text{M}}_{A+B}}\left(X\right)=\left(0.3,0.3,0.3,0.6,0.3\right),\\ \bar{\text{O}{\text{M}}_{A+B}}\left(X\right)=\left(0.5,0.4,0.5,0.6,0.5\right). \end{array}$$

Based on the model in Definition 3, we can conclude the relevant properties of optimistic multigranulation *T*-fuzzy rough sets accordingly.

## 3. Properties of Optimistic Multigranulation *T*-Fuzzy Rough Sets

In this section, we will study the properties of optimistic multigranulation *T*-fuzzy rough set which is on the rough approximation problem in a *T*-fuzzy approximation space.

**Proposition 5 .**

Let (*U*, *R*_*A*_1__, *R*_*A*_2__,…, *R*_*A*_*n*__) be a *T*-fuzzy approximation space; let *R*_*A*_*i*__, *i* ∈ {1,2, 3,…, *n*}, be the different *T*-fuzzy similarity relations, for all *x*, *y* ∈ *U*, *a*, *b* ∈ *I*, and *X*, *Y* ∈ *F*(*U*). Then, the optimistic multigranulation *T*-fuzzy lower approximation has the following properties:

1. $\underline{OM_{\sum_{i=1}^{n}A_{i}}}(X)\subseteq X$;
2. $\underline{OM_{\sum_{i=1}^{n}A_{i}}}(\underline{OM_{\sum_{i=1}^{n}A_{i}}}(X))=\underline{OM_{\sum_{i=1}^{n}A_{i}}}(X)$;
3. $\underline{OM_{\sum_{i=1}^{n}A_{i}}}(X\cap Y)\subseteq \underline{OM_{\sum_{i=1}^{n}A_{i}}}(X)\cap \underline{OM_{\sum_{i=1}^{n}A_{i}}}(Y)$;
4. $X\subseteq Y\Rightarrow \underline{OM_{\sum_{i=1}^{n}A_{i}}}(X)\subseteq \underline{OM_{\sum_{i=1}^{n}A_{i}}}(Y)$;
5. $\underline{OM_{\sum_{i=1}^{n}A_{i}}}(X\cup Y)⊇\underline{OM_{\sum_{i=1}^{n}A_{i}}}(X)\cup \underline{OM_{\sum_{i=1}^{n}A_{i}}}(Y)$;
6. $\underline{OM_{\sum_{i=1}^{n}A_{i}}}(\Theta (1_{x},\underline{\underline{a}}))(y)=\underline{OM_{\sum_{i=1}^{n}A_{i}}}(\Theta (1_{y},\underline{\underline{a}}))(x)={⋁}_{i=1}^{n}\theta (R_{A_{i}}(x,y),a)$;
7. $\underline{OM_{\sum_{i=1}^{n}A_{i}}}(\theta (1_{x},\underline{\underline{a}}))(x)=a$;
8. $\underline{OM_{\sum_{i=1}^{n}A_{i}}}(\Theta (\underline{\underline{a}},X))=\Theta (\underline{\underline{a}},\underline{OM_{\sum_{i=1}^{n}A_{i}}}(X))$;
9. $\underline{OM_{\sum_{i=1}^{n}A_{i}}}(X,\underline{\underline{a}})\subseteq \Theta (X,\underline{\underline{a}})\subseteq \Theta (\underline{OM_{\sum_{i=1}^{n}A_{i}}}(X),\underline{\underline{a}})$;
10. $\underline{OM_{\sum_{i=1}^{n}A_{i}}}(\underline{\underline{a}})=\underline{\underline{a}}$;
11. $\underline{OM_{\sum_{i=1}^{n}A_{i}}}(\underline{\underline{a}}\vee 1_{Z})(x)={⋁}_{i=1}^{n}{⋀}_{u\notin Z}\theta (R_{A_{i}}(u,x),a)$;
12. $\underline{\sum_{i=1}^{n}A_{i}}((\underline{\underline{\theta (a,b)}})\vee 1_{Z})(x)=\Theta ((\underline{\underline{a}}),\underline{OM_{\sum_{i=1}^{n}A_{i}}}(b\vee 1_{Z}))$;
13. ${⋀}_{a\in I}\theta (\underline{OM_{\sum_{i=1}^{n}A_{i}}}(\Theta (1_{x},\underline{\underline{a}}))(y),a)={⋀}_{i=1}^{n}R_{A_{i}}(x,y)$.

**Proof**

We only need to prove the proposition in a *T*-fuzzy approximation space (*U*, *R*_*A*_, *R*_*B*_) for convenience. All items hold when *R*_*A*_ = *R*_*B*_. When *R*_*A*_ ≠ *R*_*B*_, (1)–(5) can be found in [29].

(6) Firstly, we can obtain
  (9) OMA+B_(Θ(1x,a__))(y) =⋀u∈Uθ(RA(u,x),Θ(1x,a__)(u))  ∨⋀u∈Uθ(RB(u,x),Θ(1x,a__)(u)) =⋀u∈Uθ(RA(u,x),Θ(1x(u),a))  ∨⋀u∈Uθ(RB(u,x),Θ(1x(u),a)) =[θ(RA(x,y),a)∧(⋀u≠xθ(RA(u,y),θ(0,a)))]  ∨[θ(RB(x,y),a)∧(⋀u≠xθ(RB(u,y),θ(0,a)))] =[θ(RA(x,y),a)∧(⋀u≠xθ(RA(u,y),1))]  ∨[θ(RB(x,y),a)∧(⋀u≠xθ(RB(u,y),1))] =(θ(RA(x,y),a)∧1)∨(θ(RB(x,y),a)∧1) =θ(RA(x,y),a)∨θ(RB(x,y),a).

The proposition can be obtained by the symmetric and the above equation.

(7) It is easy to prove according to item (6).
(8) For any *x* ∈ *X*,
  (10) OMA+B_(Θ(a__,X))(x) =⋀u∈Uθ(RA(u,x),Θ(a__,X)(u))  ∨⋀u∈Uθ(RB(u,x),Θ(a__,X)(u)) =⋀u∈Uθ(RA(u,x),θ(a,X(u)))  ∨⋀u∈Uθ(RB(u,x),θ(a,X(u))) =⋀u∈Uθ(a,θ(RA(u,x),X(u)))  ∨⋀u∈Uθ(a,θ(RB(u,x),X(u))) =θ(a,⋀u∈Uθ(RA(u,x),X(u)))  ∨θ(a,⋀u∈Uθ(RB(u,x),X(u))) =θ(a,⋀u∈Uθ(RA(u,x),X(u))    ∨⋀u∈Uθ(RB(u,x),X(u))) =θ(a,OMA+B_(X)(x))=Θ(a__,OMA+B_(X))(x).
(9) This item follows immediately from item (1) and *θ*(3).
(10) For any *x* ∈ *X*, we have
  (11) OMA+B_(a__)(x) =⋀u∈Uθ(RA(u,x),a)∨⋀u∈Uθ(RB(u,x),a) =θ(⋁u∈URA(u,x),a)∨θ(⋁u∈URB(u,x),a) =θ(1,a)=a.
(11) For any *x* ∈ *U*, we can know
  (12) OMA+B_((a__)∨1Z)(x) =⋀u∈Uθ(RA(u,x),a∨1Z(u))  ∨⋀u∈Uθ(RB(u,x),a∨1Z(u)) =[⋀u∈Zθ(RA(u,x),1)∧⋀u∉Zθ(RA(u,x),a)]  ∨[⋀u∈Zθ(RB(u,x),1)∧⋀u∉Zθ(RB(u,x),a)] =⋀u∉Zθ(RA(u,x),a)∨⋀u∉Zθ(RB(u,x),a).
(12) For any *x* ∈ *U*, we have
  (13) OMA+B_((θ(a,b)__)∨1Z)(x) =⋀u∈Uθ(RA(u,x),(θ(a,b)__∨1Z)(u))  ∨⋀u∈Uθ(RB(u,x),(θ(a,b)__∨1Z)(u)) =[⋀u∈Zθ(RA(u,x),1)∧⋀u∉Zθ(RA(u,x),θ(a,b))]  ∨[⋀u∈Uθ(RB(u,x),1)∧⋀u∉Zθ(RB(u,x),θ(a,b))] =⋀u∉Zθ(RA(u,x),θ(a,b))∨⋀u∉Zθ(RB(u,x),θ(a,b)) =θ(a,⋀u∉Zθ(RA(u,x),b))∨θ(a,⋀u∉Zθ(RB(u,x),b)) =θ(a,⋀u∉Zθ(RA(u,x),b)∨⋀u∉Zθ(RB(u,x),b)) =θ(a,OMA+B_((b__)∨1Z)(x)).
(13) According to item (6), we can have
  (14) ⋀a∈Iθ(OMA+B_(Θ(1x,a__)(y),a)) =⋀a∈Iθ(θ(RA(x,y),a)∨θ(RB(x,y),a),a) =⋀a∈I[θ(θ(RA(x,y),a),a)∧θ(θ(RB(x,y),a),a)] =[⋀a∈Iθ(θ(RA(x,y),a),a)]  ∧[⋀a∈Iθ(θ(RB(x,y),a),a)] =RA(x,y)∧RB(x,y).

**Proposition 6 .**

Let (*U*, *R*_*A*_1__, *R*_*A*_2__,…, *R*_*A*_*n*__) be a *T*-fuzzy approximation space; let *R*_*A*_*i*__, *i* ∈ {1,2, 3,…, *n*}, be the different *T*-fuzzy similarity relations. For all *x*, *y* ∈ *U*, *a*, *b* ∈ *I*, and *X*, *Y* ∈ *F*(*U*), the optimistic multigranulation *T*-fuzzy upper approximation has the following properties:

1. $X\subseteq \bar{OM_{\sum_{i=1}^{n}A_{i}}}(X)$;
2. $\bar{OM_{\sum_{i=1}^{n}A_{i}}}(\bar{OM_{\sum_{i=1}^{n}A_{i}}}(X))=\bar{OM_{\sum_{i=1}^{n}A_{i}}}(X)$;
3. $\bar{OM_{\sum_{i=1}^{n}A_{i}}}(X\cup Y)⊇\bar{OM_{\sum_{i=1}^{n}A_{i}}}(X)\cup \bar{OM_{\sum_{i=1}^{n}A_{i}}}(Y)$;
4. $X\subseteq Y\Rightarrow \bar{OM_{\sum_{i=1}^{n}A_{i}}}(X)\subseteq \bar{OM_{\sum_{i=1}^{n}A_{i}}}(Y)$;
5. $\bar{OM_{\sum_{i=1}^{n}A_{i}}}(X\cap Y)\subseteq \bar{OM_{\sum_{i=1}^{n}A_{i}}}(X)\cap \bar{OM_{\sum_{i=1}^{n}A_{i}}}(Y)$;
6. $\bar{OM_{\sum_{i=1}^{n}A_{i}}}(1_{x})(y)=\bar{OM_{\sum_{i=1}^{n}A_{i}}}(1_{y})(x)={⋁}_{i=1}^{n}R_{A_{i}}(x,y)$;
7. $\bar{OM_{\sum_{i=1}^{n}A_{i}}}(T(\underline{\underline{a}},X))=T(\underline{\underline{a}},\bar{OM_{\sum_{i=1}^{n}A_{i}}}(X))$;
8. $\bar{OM_{\sum_{i=1}^{n}A_{i}}}(\Theta (X,\underline{\underline{a}}))⊇\Theta (X,\underline{\underline{a}})⊇\Theta (\bar{OM_{\sum_{i=1}^{n}A_{i}}}(X),\underline{\underline{a}})$;
9. $\bar{OM_{\sum_{i=1}^{n}A_{i}}}(\underline{\underline{a}})=\underline{\underline{a}}$;
10. $\bar{OM_{\sum_{i=1}^{n}A_{i}}}(1_{Z})(x)={⋀}_{i=1}^{n}{⋁}_{u\in Z}R_{A_{i}}(u,x)$;
11. $||\bar{OM_{\sum_{i=1}^{n}A_{i}}}(X)||=||X||$, where ||*X*|| = sup⁡_*u*∈*U*_ *X*(*u*).

**Proof**

We only need to prove the proposition in a *T*-fuzzy approximation space (*U*, *R*_*A*_, *R*_*B*_) for convenience. All items hold when *R*_*A*_ = *R*_*B*_. When *R*_*A*_ ≠ *R*_*B*_, (1)–(5) can be found in [29].

(6) For any *x* ∈ *U*,
  (15) OMA+B¯(1x)(y) =⋁u∈UT(RA(u,y),1x(u))∧⋁u∈UT(RB(u,y),1x(u)) =RA(x,y)∧RB(x,y).

Therefore, (6) can hold by the symmetric.

(7) For any *x* ∈ *X*,
  (16) OMA+B¯(T(a__,X))(x) =⋁u∈UT(RA(u,y),T(a__,X)(u))  ∧⋁u∈UT(RB(u,y),T(a__,X)(u)) =⋁u∈UT(RA(u,y),T(a,X(u)))  ∧⋁u∈UT(RB(u,y),T(a,X(u))) =⋁u∈UT(a,T(RA(u,y),X(u)))  ∧⋁u∈UT(a,T(RB(u,y),X(u))) =T(a,⋁u∈UT(RA(u,y),X(u)))  ∧T(a,⋁u∈UT(RB(u,y),X(u))) =T(a,⋁u∈UT(RA(u,y),X(u))    ∧⋁u∈UT(RB(u,y),X(u))) =T(a,OMA+B¯(X))(x).
(8) It can be easily proved by item (1) and *θ*(2).
(9) For any *x* ∈ *U*,
  (17) OMA+B¯(a__)(x) =⋁u∈UT(RA(u,x),a)∧⋁u∈UT(RB(u,x),a) =T(⋁u∈URA(u,x),a)∧T(⋁u∈URB(u,x),X(u)) =T(1,a) =a.  
(10) For any *Z*⊆*U*,
  (18) OMA+B¯(1Z)(x) =⋁u∈UT(RA(u,x),(1Z)(u))∧⋁u∈UT(RB(u,x),(1Z)(u)) =⋁u∈ZT(RA(u,x),1)∧⋁u∈ZT(RB(u,x),1) =⋁u∈ZRA(u,x)∧⋁u∈ZRB(u,x).
(11) Let *a* = ||*X*||, so $X\subseteq \underline{\underline{a}}$. According to items (1) and (9), we can have
  (19) $$\begin{array}{c} X\subseteq \bar{\text{O}{\text{M}}_{A+B}}\left(X\right)\subseteq \bar{\text{O}{\text{M}}_{A+B}}\left(\underline{\underline{a}}\right). \end{array}$$

Therefore, $a=||X||\leq ||\bar{\text{O}{\text{M}}_{A+B}}(X)||\leq a$.

**Proposition 7 .**

Let (*U*, *R*_*A*_1__, *R*_*A*_2__,…, *R*_*A*_*n*__) be a *T*-fuzzy approximation space; let *R*_*A*_*i*__, *i* ∈ {1,2, 3,…, *n*}, be the different *T*-fuzzy similarity relations. For all *x*, *y* ∈ *U*, *a*, *b* ∈ *I*, and *X*, *Y* ∈ *F*(*U*), the optimistic multigranulation *T*-fuzzy lower and upper approximation operators have the following properties:

1. $\underline{OM_{\sum_{i=1}^{n}A_{i}}}(X)={⋀}_{a\in I}\Theta (\bar{OM_{\sum_{i=1}^{n}A_{i}}}(\Theta (X,\underline{\underline{a}})),\underline{\underline{a}})$;
2. $\bar{OM_{\sum_{i=1}^{n}A_{i}}}(X)={⋀}_{a\in I}\Theta (\underline{OM_{\sum_{i=1}^{n}A_{i}}}(\Theta (X,\underline{\underline{a}})),\underline{\underline{a}})$;
3. $\underline{OM_{\sum_{i=1}^{n}A_{i}}}(\Theta (X,\underline{\underline{a}}))=\Theta (\bar{OM_{\sum_{i=1}^{n}A_{i}}}(X),\underline{\underline{a}})$;
4. $\underline{OM_{\sum_{i=1}^{n}A_{i}}}\Theta (\bar{OM_{\sum_{i=1}^{n}A_{i}}}(X),\underline{\underline{a}})=\Theta (\bar{OM_{\sum_{i=1}^{n}A_{i}}}(X),\underline{\underline{a}})$;
5. $\theta (X,\underline{OM_{\sum_{i=1}^{n}A_{i}}}(Y))=\theta (\bar{OM_{\sum_{i=1}^{n}A_{i}}}(X),Y)$.

**Proof**

We only need to prove the proposition in a *T*-fuzzy approximation space (*U*, *R*_*A*_, *R*_*B*_) for convenience. All items hold when *R*_*A*_ = *R*_*B*_. When *R*_*A*_ ≠ *R*_*B*_, the proposition can be proved as follows.

(1) For any *x* ∈ *U*,
  (20) ⋀a∈IΘ(OMA+B¯(Θ(X,a__)),a__)(x) =⋀a∈Iθ(⋁u∈UT(RA(u,x),θ(X(u),a))     ∧⋁u∈UT(RB(u,x),θ(X(u),a)),a) =⋀a∈Iθ(⋁u∈UT(RA(u,x),θ(X(u),a)),a)  ∨⋀a∈Iθ(⋁u∈UT(RB(u,x),θ(X(u),a)),a) =⋀a∈I ⋀u∈Uθ(RA(u,x),θ(θ(X(u),a),a))  ∨⋀a∈I ⋀u∈Uθ(RB(u,x),θ(θ(X(u),a),a)) =⋀u∈Uθ(RA(u,x),X(u))∨⋀u∈Uθ(RB(u,x),X(u)) =OMA+B_(X)(x).
(2) For any *x* ∈ *U*,
  (21) ⋀a∈IΘ(OMA+B_(Θ(X,a__)),a__) =⋀a∈Iθ(⋀u∈Uθ(RA(u,x),θ(X(u),a))     ∨⋀u∈Uθ(RB(u,x),θ(X(u),a)),a) =⋀a∈Iθ(⋀u∈Uθ(RA(u,x),θ(X(u),a)),a)  ∧⋀a∈Iθ(⋀u∈Uθ(RB(u,x),θ(X(u),a)),a) =⋀a∈Iθ(⋀u∈Uθ(T(RA(u,x),X(u)),a),a)  ∧⋀a∈Iθ(⋀u∈Uθ(T(RB(u,x),X(u)),a),a) =⋀a∈Iθ(θ(⋁u∈UT(RA(u,x),X(u)),a),a)  ∧⋀a∈Iθ(θ(⋁u∈UT(RB(u,x),X(u)),a),a) =⋁u∈UT(RA(u,x),X(u))∧⋁u∈UT(RB(u,x),X(u)) =OMA+B¯(X)(x).
(3) For any *x* ∈ *U*,
  (22) OMA+B_(Θ(X,a__))(x) =⋀a∈Iθ(RA(u,x),θ(X(u),a))  ∨⋀a∈Iθ(RB(u,x),θ(X(u),a)) =⋀a∈Iθ(T(RA(u,x),X(u)),a)  ∨⋀a∈Iθ(T(RB(u,x),X(u)),a) =θ(⋁a∈IT(RA(u,x),X(u)),a)  ∨θ(⋁a∈IT(RB(u,x),X(u)),a) =θ(⋁a∈IT(RA(u,x),X(u))    ∧⋁a∈IT(RB(u,x),X(u)),a) =θ(OMA+B¯(X)(x),a).
(4) We can have, by item (3) in Proposition 7 and item (2) in Proposition 6,
  (23) OMA+B_(Θ(OMA+B¯(X),a__)) =Θ(OMA+B¯(OMA+B¯(X)),a__)=Θ(OMA+B¯(X),a__).
(5) According to *θ*6 and *θ*9, we can have
  (24) θ(X,OMA+B_(Y)) =⋀u∈Uθ(X(u),OMA+B_(Y)(u)) =⋀u∈Uθ(X(u),⋀v∈Uθ(RA(v,u),Y(v))     ∨⋀v∈Uθ(RB(v,u),Y(v))) =⋀u∈Uθ(X(u),⋀v∈Uθ(RA(v,u),Y(v)))  ∨⋀u∈Uθ(X(u),⋀v∈Uθ(RB(v,u),Y(v))) =⋀u∈U ⋀v∈Uθ(T(RA(v,u),X(u)),Y(v))  ∨⋀u∈U ⋀v∈Uθ(T(RB(v,u),X(u)),Y(v)) =⋀v∈Uθ(⋁u∈UT(RA(v,u),X(u)),Y(v))  ∨⋀v∈Uθ(⋁u∈UT(RB(v,u),X(u)),Y(v)) =⋀v∈Uθ(⋁u∈UT(RA(v,u),X(u))     ∧⋁u∈UT(RB(v,u),X(u)),Y(v)) =⋀v∈Uθ(OMA+B¯(X)(v),Y(v)).

## 4. Model and Properties of Pessimistic Multigranulation *T*-Fuzzy Rough Sets

In Sections 2 and 3, we introduced the model and properties of optimistic multigranulation *T*-fuzzy rough sets. Now, we begin to study a new kind of multigranulation *T*-fuzzy rough sets called the pessimistic multigranulation rough set in the *T*-fuzzy approximation space.

**Definition 8 .**

Let (*U*, *R*_*A*_1__, *R*_*A*_2__,…, *R*_*A*_*n*__) be a *T*-fuzzy approximation space. For any *X* ∈ *F*(*U*), we can define the pessimistic multigranulation *T*-fuzzy lower and upper approximations of *X* as follows:

(25) $$\begin{array}{c} \underline{\text{P}{\text{M}}_{\sum_{i=1}^{n}A_{i}}}\left(X\right)\left(x\right)=\;\;\overset{n}{\underset{i=1}{⋀}}\left(\underset{u\in U}{⋀}\theta \left(R_{A_{i}}\left(u,x\right),X\left(u\right)\right)\right),\\ \;\;\bar{\text{P}{\text{M}}_{\sum_{i=1}^{n}A_{i}}}\left(X\right)\left(x\right)=\;\;\overset{n}{\underset{i=1}{⋁}}\left(\underset{u\in U}{⋁}T\left(R_{A_{i}}\left(u,x\right),X\left(u\right)\right)\right), \end{array}$$

where *“*⋁” means “max,” *“*⋀” means “min,” and *θ* and *T* are defined in Section 2. $\underline{\text{P}{\text{M}}_{\sum_{i=1}^{n}A_{i}}}$ and $\bar{\text{P}{\text{M}}_{\sum_{i=1}^{n}A_{i}}}$ are referred to as the pessimistic multigranulation *T*-fuzzy lower and *T* upper approximation operators. The pair $\left(\underline{\text{P}{\text{M}}_{\sum_{i=1}^{n}A_{i}}}(X),\bar{\text{P}{\text{M}}_{\sum_{i=1}^{n}A_{i}}}(X)\right)$ is called the pessimistic multigranulation *T*-fuzzy rough set of *X*. If $\underline{\text{P}{\text{M}}_{\sum_{i=1}^{n}A_{i}}}(X)=\bar{\text{P}{\text{M}}_{\sum_{i=1}^{n}A_{i}}}(X)$, then *X* is referred to as pessimistic definable under the *T*-fuzzy approximation space; otherwise, *X* is referred to as pessimistic undefinable. The boundary of the pessimistic multigranulation *T*-fuzzy rough set *X* is defined as

(26) $$\begin{array}{c} {\text{Bnd}}_{\sum_{i=1}^{n}A_{i}}^{P}\left(X\right)=\bar{{\text{PM}}_{\sum_{i=1}^{n}A_{i}}}\left(X\right)\cap \left(\sim \underline{{\text{PM}}_{\sum_{i=1}^{n}A_{i}}}\left(X\right)\right). \end{array}$$

**Example 9 (continued from Example 4).**

From Definition 8, we can compute pessimistic multigranulation lower and upper approximations of *X* over the *T*-fuzzy similar relations *R*_*A*_ and *R*_*B*_ as

(27) $$\begin{array}{c} \underline{{\text{PM}}_{A+B}}=\left\{0.3,0.3,0.3,0.3,0.3\right\},\\ \bar{{\text{PM}}_{A+B}}=\left\{0.5,0.5,0.5,0.6,0.6\right\}. \end{array}$$

From the definition of the pessimistic multigranulation *T*-fuzzy lower and upper approximations, it is possible to deduce the following properties of the pessimistic multigranulation *T*-fuzzy lower and upper approximation operators.

**Proposition 10 .**

Let (*U*, *R*_*A*_1__, *R*_*A*_2__,…, *R*_*A*_*n*__) be a *T*-fuzzy approximation space; let *R*_*A*_*i*__  (*i* ∈ {1,2, 3,…, *n*}) be the different *T*-fuzzy similarity relations. For all *x*, *y* ∈ *U*, *a*, *b* ∈ *I*, and *X*, *Y* ∈ *F*(*U*), the pessimistic multigranulation *T*-fuzzy lower approximation has the following properties:

1. $\underline{PM_{\sum_{i=1}^{n}A_{i}}}(X)\subseteq X$;
2. $\underline{PM_{\sum_{i=1}^{n}A_{i}}}(\underline{PM_{\sum_{i=1}^{n}A_{i}}}(X))\subseteq \underline{PM_{\sum_{i=1}^{n}A_{i}}}(X)$;
3. $\underline{PM_{\sum_{i=1}^{n}A_{i}}}(X\cap Y)=\underline{PM_{\sum_{i=1}^{n}A_{i}}}(X)\cap \underline{PM_{\sum_{i=1}^{n}A_{i}}}(Y)$;
4. $X\subseteq Y\Rightarrow \underline{PM_{\sum_{i=1}^{n}A_{i}}}(X)\subseteq \underline{PM_{\sum_{i=1}^{n}A_{i}}}(Y)$;
5. $\underline{PM_{\sum_{i=1}^{n}A_{i}}}(X\cup Y)⊇\underline{PM_{\sum_{i=1}^{n}A_{i}}}(X)\cup \underline{PM_{\sum_{i=1}^{n}A_{i}}}(Y)$;
6. $\underline{PM_{\sum_{i=1}^{n}A_{i}}}(\Theta (1_{x},\underline{\underline{a}}))(y)=\underline{PM_{\sum_{i=1}^{n}A_{i}}}(\Theta (1_{y},\underline{\underline{a}}))(x)={⋁}_{i=1}^{n}\theta (R_{A_{i}}(x,y),a)$;
7. $\underline{PM_{\sum_{i=1}^{n}A_{i}}}(\theta (1_{x},\underline{\underline{a}}))(x)=a$;
8. $\underline{PM_{\sum_{i=1}^{n}A_{i}}}(\Theta (\underline{\underline{a}},X))=\Theta (\underline{\underline{a}},\underline{PM_{\sum_{i=1}^{n}A_{i}}}(X))$;
9. $\underline{PM_{\sum_{i=1}^{n}A_{i}}}(X,\underline{\underline{a}})\subseteq \Theta (X,\underline{\underline{a}})\subseteq \Theta (\underline{PM_{\sum_{i=1}^{n}A_{i}}}(X),\underline{\underline{a}})$;
10. $\underline{PM_{\sum_{i=1}^{n}A_{i}}}(\underline{\underline{a}})=\underline{\underline{a}}$;
11. $\underline{PM_{\sum_{i=1}^{n}A_{i}}}((\underline{\underline{a}})\vee 1_{Z})(x)={⋁}_{i=1}^{n}{⋀}_{u\notin Z}\theta (R_{A_{i}}(u,x),a)$;
12. $\underline{\sum_{i=1}^{n}A_{i}}((\underline{\underline{\theta (a,b)}})\vee 1_{Z})(x)=\Theta ((\underline{\underline{a}}),\underline{PM_{\sum_{i=1}^{n}A_{i}}}(b\vee 1_{Z}))$;
13. ${⋀}_{a\in I}\theta (\underline{PM_{\sum_{i=1}^{n}A_{i}}}(\Theta (1_{x},\underline{\underline{a}}))(y),a)={⋀}_{i=1}^{n}R_{A_{i}}(x,y)$.

**Proof**

We only need to prove the proposition in a *T*-fuzzy approximation space (*U*, *R*_*A*_, *R*_*B*_) for convenience. All items hold when *R*_*A*_ = *R*_*B*_. When *R*_*A*_ ≠ *R*_*B*_, the proposition can be proved as follows.

(1) For any *x* ∈ *U*,
  (28) PMA+B_(X)(x) =⋀u∈Uθ(RA(u,x),X(u))∧⋀u∈Uθ(RB(u,x),X(u)) ≤θ(RA(x,x),X(x))∧θ(RB(x,x),X(x))=X(x).
(2) According to item (1), it obviously holds.
(3) For any *x* ∈ *U*,
  (29) PMA+B_(X∩Y)(x) =⋀u∈Uθ(RA(u,x),X(u)∧Y(u))  ∧⋀u∈Uθ(RB(u,x),X(u)∧Y(u)) =[⋀u∈Uθ(RA(u,x),X(u))∧⋀u∈Uθ(RA(u,x),Y(u))]  ∧[⋀u∈Uθ(RB(u,x),X(u))∧⋀u∈Uθ(RB(u,x),Y(u))] =[⋀u∈Uθ(RA(u,x),X(u))∧⋀u∈Uθ(RB(u,x),X(u))]  ∧[⋀u∈Uθ(RA(u,x),Y(u))∧⋀u∈Uθ(RB(u,x),Y(u))] =PMA+B_(X)(x)∩PMA+B_(Y)(x).
(4) For any *x* ∈ *U*, we have *X*(*x*) ≤ *Y*(*x*) by *X*⊆*Y*. So
  (30) PMA+B_(X)(x) =⋀u∈Uθ(RA(u,x),X(u))∧⋀u∈Uθ(RB(u,x),X(u)) ≤⋀u∈Uθ(RA(u,x),Y(u))∧⋀u∈Uθ(RB(u,x),Y(u)) =PMA+B_(Y)(x).
(5) It is easy to prove according to item (4).
(6) First of all, we have
  (31) PMA+B_(Θ(1x,a__))(y) =⋀u∈Uθ(RA(u,y),(Θ(1x,a__)(u)))  ∧⋀u∈Uθ(RB(u,y),(Θ(1x,a__)(u))) =⋀u∈Uθ(RA(u,y),(θ(1x(u),a)))  ∧⋀u∈Uθ(RB(u,y),(θ(1x(u),a))) =⋀u=xθ(RA(x,y),(θ(1,a)))  ∧⋀u=xθ(RB(x,y),(θ(1,a))) =θ(RA(x,y),a)∧θ(RB(x,y),a).

By the symmetric and the above equation, item (6) can be proved.

(7) It can be verified by item (6).
(8) For any *x* ∈ *U*,
  (32) PMA+B_(Θ(a__,X))(x) =⋀u∈Uθ(RA(u,x),Θ(a__,X)(u))  ∧⋀u∈Uθ(RB(u,x),Θ(a__,X)(u)) =⋀u∈Uθ(RA(u,x),θ(a,X(u)))  ∧⋀u∈Uθ(RB(u,x),θ(a,X(u))) =⋀u∈Uθ(a,θ(RA(u,x),X(u)))  ∧⋀u∈Uθ(a,θ(RB(u,x),X(u))) =θ(a,⋀u∈Uθ(RA(u,x),X(u)))  ∧⋀u∈Uθ(RB(u,x),X(u)) =Θ(a,PMA+B_(X))(x).
(9) It is easy to prove by item (1) and *θ*3.
(10) For any *x* ∈ *U*,
  (33) PMA+B_(a__)(x)=⋀u∈Uθ(RA(u,x),a)∧⋀u∈Uθ(RB(u,x),a)=θ(⋁u∈URA(u,x),a)∧θ(⋁u∈URB(u,x),a)=θ(1,a)=a.
(11) For any *x* ∈ *U*,
  (34) PMA+B_((a__)∨1Z)(x) =⋀u∈Uθ(RA(u,x),((a__)∨1Z)(u))  ∧⋀u∈Uθ(RB(u,x),((a__)∨1Z)(u)) =⋀u∈Uθ(RA(u,x),a∨1Z(u))  ∧⋀u∈Uθ(RB(u,x),a∨1Z(u)) =⋀u∈Zθ(RA(u,x),1)∨⋀u∉Zθ(RA(u,x),a)  ∨⋀u∈Zθ(RB(u,x),1)∨⋀u∉Zθ(RB(u,x),a) =⋀u∉Zθ(RA(u,x),a)∨⋀u∉Zθ(RB(u,x),a).
(12) For any *x* ∈ *U*,
  (35) PMA+B_((θ(a,b)__)∨1Z)(x) =⋀u∈Uθ(RA(u,x),((θ(a,b)__)∨1Z)(u))  ∧⋀u∈Uθ(RB(u,x),((θ(a,b)__)∨1Z)(u)) =⋀u∈Uθ(RA(u,x),(θ(a,b)∨1Z(u)))  ∧⋀u∈Uθ(RB(u,x),(θ(a,b)∨1Z(u))) =⋀u∉Zθ(RA(u,x),θ(a,b))∧⋀u∉Zθ(RB(u,x),θ(a,b)) =⋀u∉Zθ(a,θ(RA(u,x),b))∧⋀u∉Zθ(a,θ(RB(u,x),b)) =θ(a,⋀u∉Zθ(RA(u,x),b)∧⋀u∉Zθ(RB(u,x),b)) =θ(a,PMA+B_((b__)∨1Z))(x).
(13) We can have, by item (6),
  (36) ⋀a∈Iθ(PMA+B_(Θ(1x,a__))(y),a) =⋀a∈Iθ(θ(RA(x,y),a)∧θ(RB(x,y),a),a) ≥⋀a∈Iθ(θ(RA(x,y),a),a)∧⋀a∈Iθ(θ(RB(x,y),a),a) =RA(x,y)∧RB(x,y).

**Proposition 11 .**

Let (*U*, *R*_*A*_1__, *R*_*A*_2__,…, *R*_*A*_*n*__) be a *T*-fuzzy approximation space; let *R*_*A*_*i*__  (*i* ∈ {1,2, 3,…, *n*}) be the different *T*-fuzzy similarity relations. For all *x*, *y* ∈ *U*, *a*, *b* ∈ *I*, and *X*, *Y* ∈ *F*(*U*), the pessimistic multigranulation *T*-fuzzy upper approximation has the following properties:

1. $X\subseteq \bar{PM_{\sum_{i=1}^{n}A_{i}}}(X)$;
2. $\bar{PM_{\sum_{i=1}^{n}A_{i}}}(\bar{PM_{\sum_{i=1}^{n}A_{i}}}(X))⊇\bar{PM_{\sum_{i=1}^{n}A_{i}}}(X)$;
3. $\bar{PM_{\sum_{i=1}^{n}A_{i}}}(X\cup Y)=\bar{PM_{\sum_{i=1}^{n}A_{i}}}(X)\cup \bar{PM_{\sum_{i=1}^{n}A_{i}}}(Y)$;
4. $X\subseteq Y\Rightarrow \bar{PM_{\sum_{i=1}^{n}A_{i}}}(X)\subseteq \bar{PM_{\sum_{i=1}^{n}A_{i}}}(Y)$;
5. $\bar{PM_{\sum_{i=1}^{n}A_{i}}}(X\cap Y)\subseteq \bar{PM_{\sum_{i=1}^{n}A_{i}}}(X)\cap \bar{PM_{\sum_{i=1}^{n}A_{i}}}(Y)$;
6. $\bar{PM_{\sum_{i=1}^{n}A_{i}}}(1_{x})(y)=\bar{PM_{\sum_{i=1}^{n}A_{i}}}(1_{y})(x)={⋁}_{i=1}^{n}R_{A_{i}}(x,y)$;
7. $\bar{PM_{\sum_{i=1}^{n}A_{i}}}(T(\underline{\underline{a}},X))=T(\underline{\underline{a}},\bar{PM_{\sum_{i=1}^{n}A_{i}}}(X))$;
8. $\bar{PM_{\sum_{i=1}^{n}A_{i}}}(\Theta (X,\underline{\underline{a}}))⊇\Theta (X,\underline{\underline{a}})⊇\Theta (\bar{PM_{\sum_{i=1}^{n}A_{i}}}(X),\underline{\underline{a}})$;
9. $\bar{PM_{\sum_{i=1}^{n}A_{i}}}(\underline{\underline{a}})=\underline{\underline{a}}$;
10. $\bar{PM_{\sum_{i=1}^{n}A_{i}}}(1_{Z})(x)={⋀}_{i=1}^{n}{⋁}_{u\in Z}R_{A_{i}}(u,x)$;
11. $||\bar{PM_{\sum_{i=1}^{n}A_{i}}}(X)||=||X||$, where ||*X*|| = sup⁡_*u*∈*U*_ *X*(*u*).

**Proof**

We only need to prove the proposition in a *T*-fuzzy approximation space (*U*, *R*_*A*_, *R*_*B*_) for convenience. All items hold when *R*_*A*_ = *R*_*B*_. When *R*_*A*_ ≠ *R*_*B*_, the proposition can be proved as follows.

(1) For any *x* ∈ *U*,
  (37) PMA+B¯(X)(x) =⋁u∈UT(RA(u,x),X(u))∨⋁u∈UT(RB(u,x),X(u)) ≥T(RA(x,x),X(x))∨T(RB(x,x),X(x))=X(x).
(2) This item can be proved by item (1).
(3) For any *x* ∈ *U*,
  (38) PMA+B¯(X∪Y)(x) =⋁u∈UT(RA(u,x),X(u)∨Y(u))  ∨⋁u∈UT(RB(u,x),X(u)∨Y(u)) =⋁u∈UT(RA(u,x),X(u))∨⋁u∈UT(RB(u,x),X(u))  ∨⋁u∈UT(RA(u,x),Y(u))∨⋁u∈UT(RB(u,x),Y(u)) =PMA+B¯(X)(x)∪PMA+B¯(Y)(x).
(4) Since *X*⊆*Y*, for any *x* ∈ *X*, we can have *X*(*x*) ≤ *Y*(*x*). Thus,
  (39) PMA+B¯(X)(x) =⋁u∈UT(RA(u,x),X(u))∨⋁u∈UT(RB(u,x),X(u)) ≤⋁u∈UT(RA(u,x),Y(u))∨⋁u∈UT(RB(u,x),Y(u)) =PMA+B¯(Y)(x).
(5) It is easy to prove by item (4).
(6) According to Definition 8, we have
  (40) PMA+B¯(1x)(y) =⋁u∈UT(RA(u,y),(1x)(u))∨⋁u∈UT(RB(u,y),(1x)(u)) =T(RA(x,y),1)∨T(RB(x,y),1) =RA(x,y)∨RB(x,y).

We can conclude that $\bar{{\text{PM}}_{A+B}}(1_{x})(y)=\bar{{\text{PM}}_{A+B}}(1_{y})(x)=R_{A}(x,y)\vee R_{B}(x,y)$ by the symmetric and the above equation.

(7) For any *x* ∈ *U*,
  (41) PMA+B¯(T(a__,X))(x) =⋁u∈UT(RA(u,x),(T(a__,X))(u))  ∨⋁u∈UT(RB(u,x),(T(a__,X))(u)) =⋁u∈UT(RA(u,x),T(a,X(u)))  ∨⋁u∈UT(RB(u,x),T(a,X(u))) =T(a,⋁u∈UT(RA(u,x),X(u)))  ∨T(a,⋁u∈UT(RB(u,x),X(u))) =T(a,⋁u∈UT(RA(u,x),X(u))    ∨⋁u∈UT(RB(u,x),X(u))) =T(a__,PMA+B¯(X))(x).
(8) It directly follows from item (1) and *θ*3.
(9) For any *x* ∈ *U*,
  (42) PMA+B¯(a__)(x) =⋁u∈UT(RA(u,x),a)∨⋁u∈UT(RB(u,x),a) =T(⋁u∈URA(u,x),a)∨T(⋁u∈URB(u,x),a)=a.
(10) For any *x* ∈ *U*,
  (43) PMA+B¯(1Z)(x) =⋁u∈UT(RA(u,x),X(u))∨⋁u∈UT(RB(u,x),X(u)) =⋁u∈ZT(RA(u,x),X(u))∨⋁u∈ZT(RB(u,x),X(u)) =⋁u∈ZRA(u,x)∨⋁u∈ZRB(u,x).
(11) Let *a* = ||*X*||, so $X\subseteq \underline{\underline{a}}$. According to items (1) and (9), we can have
  (44) $$\begin{array}{c} X\subseteq \bar{{\text{PM}}_{A+B}}\left(X\right)\subseteq \bar{{\text{PM}}_{A+B}}\left(\underline{\underline{a}}\right). \end{array}$$

Therefore, $a=||X||\leq ||\bar{{\text{PM}}_{A+B}}(X)||\leq a$.

**Proposition 12 .**

Let (*U*, *R*_*A*_1__, *R*_*A*_2__,…, *R*_*A*_*n*__) be a *T*-fuzzy approximation space, and let *R*_*A*_*i*__  (*i* ∈ {1,2, 3,…, *n*}) be the different *T*-fuzzy similarity relations. For all *x*, *y* ∈ *U*, *a*, *b* ∈ *I*, and *X*, *Y* ∈ *F*(*U*), the pessimistic multigranulation *T*-fuzzy lower and upper approximation operators have the following properties:

1. ${⋀}_{a\in I}\Theta (\bar{PM_{\sum_{i=1}^{n}A_{i}}}(\Theta (X,\underline{\underline{a}})),\underline{\underline{a}})=\underline{PM_{\sum_{i=1}^{n}A_{i}}}(X)$;
2. ${⋀}_{a\in I}\Theta (\underline{PM_{\sum_{i=1}^{n}A_{i}}}(\Theta (X,\underline{\underline{a}})),\underline{\underline{a}})=\bar{PM_{\sum_{i=1}^{n}A_{i}}}(X)$;
3. $\underline{PM_{\sum_{i=1}^{n}A_{i}}}(\Theta (X,\underline{\underline{a}}))=\Theta (\bar{PM_{\sum_{i=1}^{n}A_{i}}}(X),\underline{\underline{a}})$;
4. $\theta (X,\underline{PM_{\sum_{i=1}^{n}A_{i}}}(Y))=\theta (\bar{PM_{\sum_{i=1}^{n}A_{i}}}(X),Y)$.

**Proof**

We only need to prove the proposition in a *T*-fuzzy approximation space (*U*, *R*_*A*_, *R*_*B*_) for convenience. All items hold when *R*_*A*_ = *R*_*B*_. When *R*_*A*_ ≠ *R*_*B*_, the proposition can be proved as follows.

(1) For any *x* ∈ *U*,
  (45) ⋀a∈IΘ(PMA+B¯(Θ(X,a__)),a__)(x) =⋀a∈Iθ(⋁u∈UT(RA(u,x),θ(X(u),a))     ∨⋁u∈UT(RB(u,x),θ(X(u),a)),a) =⋀a∈Iθ(⋁u∈UT(RA(u,x),θ(X(u),a)),a)  ∧⋀a∈Iθ(⋁u∈UT(RB(u,x),θ(X(u),a)),a) =⋀a∈I ⋀u∈Uθ(RA(u,x),θ(θ(X(u),a),a))  ∧⋀a∈I ⋀u∈Uθ(RB(u,x),θ(θ(X(u),a),a)) =⋀u∈Uθ(RA(u,x),X(u))∧⋀u∈Uθ(RB(u,x),X(u)) =PMA+B_(X)(x).
(2) For any *x* ∈ *U*,
  (46) ⋀a∈IΘ(PMA+B_(Θ(X,a__)),a__) =⋀a∈Iθ(⋀u∈Uθ(RA(u,x),θ(X(u),a)))  ∧⋀u∈Uθ(RB(u,x),θ(X(u),a)),a =⋀a∈Iθ(⋀u∈Uθ(RA(u,x),θ(X(u),a)),a)  ∨⋀a∈Iθ(⋀u∈Uθ(RB(u,x),θ(X(u),a)),a) =⋀a∈Iθ(⋀u∈Uθ(T(RA(u,x),X(u)),a),a)  ∨⋀a∈Iθ(⋀u∈Uθ(T(RB(u,x),X(u)),a),a) =⋀a∈Iθ(θ(⋁u∈UT(RA(u,x),X(u)),a),a)  ∨⋀a∈Iθ(θ(⋁u∈UT(RB(u,x),X(u)),a),a) =⋁u∈UT(RA(u,x),X(u))∨⋁u∈UT(RB(u,x),X(u)) =PMA+B¯(X)(x).
(3) For any *x* ∈ *U*,
  (47) PMA+B_(Θ(X,a__))(x) =⋀a∈Iθ(RA(u,x),θ(X(u),a))  ∧⋀a∈Iθ(RB(u,x),θ(X(u),a)) =⋀a∈Iθ(T(RA(u,x),X(u)),a)  ∧⋀a∈Iθ(T(RB(u,x),X(u)),a) =θ(⋁a∈IT(RA(u,x),X(u)),a)  ∧θ(⋁a∈IT(RB(u,x),X(u)),a) =θ(⋁a∈IT(RA(u,x),X(u))∨⋁a∈IT(RB(u,x),X(u)),a) =θ(PMA+B¯(X)(x),a).
(4) According to *θ*6 and *θ*9, we can obtain
  (48) θ(X,PMA+B_(Y)) =⋀u∈Uθ(X(u),PMA+B_(Y)(u)) =⋀u∈Uθ(X(u),⋀v∈Uθ(RA(v,u),Y(v))     ∧⋀v∈Uθ(RB(v,u),Y(v))) =⋀u∈Uθ(X(u),⋀v∈Uθ(RA(v,u),Y(v)))  ∧⋀u∈Uθ(X(u),⋀v∈Uθ(RB(v,u),Y(v))) =⋀u∈U ⋀v∈Uθ(T(RA(v,u),X(u)),Y(v))  ∧⋀u∈U ⋀v∈Uθ(T(RB(v,u),X(u)),Y(v)) =⋀v∈Uθ(⋁u∈UT(RA(v,u),X(u)),Y(v))  ∧⋀v∈Uθ(⋁u∈UT(RB(v,u),X(u)),Y(v)) =⋀v∈Uθ(⋁u∈UT(RA(v,u),X(u)))  ∨⋁u∈UT(RB(v,u),X(u)),Y(v) =⋀v∈Uθ(PMA+B¯(X)(v)),Y(v).

Then, this proposition is proved.

## 5. Relationships between Multigranulation and Classical *T*-Fuzzy Rough Sets

Based on the *T*-fuzzy similarity relation, after the discussion about the properties of the optimistic and pessimistic multigranulation *T*-fuzzy rough sets, we will investigate the relationships among the two types of multigranulation *T*-fuzzy rough sets and the classical *T*-fuzzy rough set in this section.

By the definitions of the optimistic and pessimistic multigranulation *T*-fuzzy rough set operators, for all *X* ∈ *F*(*U*), the relationship can be easily obtained as

(49) PM∑i=1nAi_(X)⊆OM∑i=1nAi_(X)⊆X⊆OM∑i=1nAi¯(X)⊆PM∑i=1nAi¯(X).

Note that if (*U*, *R*) is a *T*-fuzzy approximation space, then $\underline{{\text{PM}}_{\sum_{i=1}^{n}A_{i}}}(X)=\underline{{\text{OM}}_{\sum_{i=1}^{n}A_{i}}}(X)=\underline{R}(X)$ and $\bar{{\text{OM}}_{\sum_{i=1}^{n}A_{i}}}(X)=\bar{{\text{PM}}_{\sum_{i=1}^{n}A_{i}}}(X)=\bar{R}(X)$. So in the special case of a *T*-fuzzy approximation space, both optimistic and pessimistic *T*-fuzzy lower and upper approximations can degenerate into the standard *T*-fuzzy lower and upper approximations.

**Proposition 13 .**

Let (*U*, *R*_*A*_1__, *R*_*A*_2__,…, *R*_*A*_*n*__) be *T*-fuzzy approximation space, and let *R*_*A*_*i*__  (*i* ∈ {1,2, 3,…, *n*}) be the different *T* fuzzy similarity relations. For all *x*, *y* ∈ *U*, *a*, *b* ∈ *I*, and *X*, *Y* ∈ *F*(*U*), one has the following:

1. $\bar{OM_{\sum_{i=1}^{n}A_{i}}}(\underline{PM_{\sum_{i=1}^{n}A_{i}}}(X))=\underline{PM_{\sum_{i=1}^{n}A_{i}}}(X)$;
2. $\underline{OM_{\sum_{i=1}^{n}A_{i}}}(\bar{PM_{\sum_{i=1}^{n}A_{i}}}(X))=\bar{PM_{\sum_{i=1}^{n}A_{i}}}(X)$;
3. $\bar{PM_{\sum_{i=1}^{n}A_{i}}}(\underline{PM_{\sum_{i=1}^{n}A_{i}}}(X))\subseteq \underline{OM_{\sum_{i=1}^{n}A_{i}}}(X)$;
4. $\underline{PM_{\sum_{i=1}^{n}A_{i}}}(\bar{PM_{\sum_{i=1}^{n}A_{i}}}(X))⊇\bar{OM_{\sum_{i=1}^{n}A_{i}}}(X)$;
5. $\theta (\bar{PM_{\sum_{i=1}^{n}A_{i}}}(X),\underline{OM_{\sum_{i=1}^{n}A_{i}}}(Y))=\theta (\bar{PM_{\sum_{i=1}^{n}A_{i}}}(X),Y)$;
6. $\theta (\bar{OM_{\sum_{i=1}^{n}A_{i}}}(X),\bar{PM_{\sum_{i=1}^{n}A_{i}}}(Y))=\theta (X,\bar{PM_{\sum_{i=1}^{n}A_{i}}}(Y))$;
7. $\underline{OM_{\sum_{i=1}^{n}A_{i}}}(\Theta (\underline{\underline{a}},\bar{PM_{\sum_{i=1}^{n}A_{i}}}(X)))=\Theta (\underline{\underline{a}},\bar{PM_{\sum_{i=1}^{n}A_{i}}}(X))$.

**Proof**

We only need to prove the proposition in a *T*-fuzzy approximation space (*U*, *R*_*A*_, *R*_*B*_) for convenience. All items hold when *R*_*A*_ = *R*_*B*_. When *R*_*A*_ ≠ *R*_*B*_, the proposition can be proved as follows.

(1) For any *x* ∈ *U*,
  (50) OMA+B¯(PMA+B_(X))(x) =⋁u∈UT(RA(u,x),PMA+B_X(u))  ∧⋁u∈UT(RB(u,x),PMA+B_X(u)) =[⋁u∈UT(RA(u,x),⋀v∈Uθ(RA(v,u),X(v))      ∧⋀v∈Uθ(RB(v,u),X(v)))]  ∧[⋁u∈UT(RB(u,x),⋀v∈Uθ(RA(v,u),X(v))       ∧⋀v∈Uθ(RB(v,u),X(v)))] ≤⋁u∈UT(RA(u,x),⋀v∈Uθ(RA(v,u),X(v)))  ∧⋁u∈UT(RB(u,x),⋀v∈Uθ(RB(v,u),X(v))) =⋁u∈U ⋀v∈UT(RA(u,x),θ(RA(v,u),X(v)))  ∧⋁u∈U ⋀v∈UT(RB(u,x),θ(RB(v,u),X(v))) ≤⋁u∈U ⋀v∈UT(RA(u,x),      θ(T(RA(v,x),RA(x,u)),X(v)))  ∧⋁u∈U ⋀v∈UT(RB(u,x),       θ(T(RB(v,x),RB(x,u)),X(v))) ≤⋀v∈Uθ(RA(v,x),X(v))∧⋀v∈Uθ(RB(v,x),X(v)) =PMA+B_(X)(x).

On the other hand, $\underline{{\text{PM}}_{A+B}}(X)\subseteq \bar{{\text{OM}}_{A+B}}(\underline{{\text{PM}}_{A+B}}(X))$. Therefore, $\underline{{\text{PM}}_{A+B}}(X)=\bar{{\text{OM}}_{A+B}}(\underline{{\text{PM}}_{A+B}}(X))$.

(2) For any *x* ∈ *U*,
  (51) OMA+B_(PMA+B¯(X))(x) =⋀u∈Uθ(RA(u,x),PMA+B¯X(u))  ∨⋀u∈Uθ(RB(u,x),PMA+B¯X(u)) =⋀u∈Uθ(RA(u,x),⋁v∈UT(RA(v,u),X(v))      ∨⋁v∈UT(RB(v,u),X(v)))  ∨⋀u∈Uθ(RB(u,x),⋁v∈UT(RA(v,u),X(v))      ∨⋁v∈UT(RB(v,u),X(v))) ≥⋀u∈Uθ(RA(u,x),⋁v∈UT(RA(v,u),X(v)))  ∨⋀u∈Uθ(RB(u,x),⋁v∈UT(RB(v,u),X(v))) =⋀u∈U ⋁v∈Uθ(RA(u,x),T(RA(v,u),X(v)))  ∨⋀u∈U ⋁v∈Uθ(RB(u,x),T(RB(v,u),X(v))) ≥⋁u∈U ⋀v∈Uθ(RA(u,x),      T(T(RA(v,x),RA(x,u)),X(v)))  ∨⋁u∈U ⋀v∈Uθ(RB(u,x),       T(T(RB(v,x),RB(x,u)),X(v))) ≥⋁v∈UT(RA(v,x),X(v))∨⋁v∈UT(RB(v,x),X(v)) =PMA+B¯(X)(x).

On the other hand, $\underline{\text{O}{\text{M}}_{A+B}}(\bar{{\text{PM}}_{A+B}}(X))\subseteq \bar{{\text{PM}}_{A+B}}(X)$. Therefore, $\underline{{\text{OM}}_{A+B}}(\bar{{\text{PM}}_{A+B}}(X))=\bar{{\text{PM}}_{A+B}}(X)$.

(3) For any *x* ∈ *U*,
  (52) PMA+B¯(PMA+B_(X))(x) =⋁u∈UT(RA(u,x),PMA+B_X(u))  ∨⋁u∈UT(RB(u,x),PMA+B_X(u)) =⋁u∈UT(RA(u,x),⋀v∈Uθ(RA(v,u),X(v))     ∧⋀v∈Uθ(RB(v,u),X(v)))  ∨⋁u∈UT(RB(u,x),⋀v∈Uθ(RA(v,u),X(v))      ∧⋀v∈Uθ(RB(v,u),X(v))) ≤⋁u∈UT(RA(u,x),⋀v∈Uθ(RA(v,u),X(v)))  ∨⋁u∈UT(RB(u,x),⋀v∈Uθ(RB(v,u),X(v))) =⋁u∈U ⋀v∈UT(RA(u,x),θ(RA(v,u),X(v)))  ∨⋁u∈U ⋀v∈UT(RB(u,x),θ(RB(v,u),X(v))) ≤⋁u∈U ⋀v∈UT(RA(u,x),      θ(T(RA(v,x),RA(x,u)),X(v)))  ∨⋁u∈U ⋀v∈UT(RB(u,x),       θ(T(RB(v,x),RB(x,u)),X(v))) ≤⋀v∈Uθ(RA(v,x),X(v))∨⋀v∈Uθ(RB(v,x),X(v)) =OMA+B_(X)(x).  
(4) For any *x* ∈ *U*,
  (53) PMA+B_(PMA+B¯(X))(x) =⋀u∈Uθ(RA(u,x),PMA+B¯(X)(u))  ∧⋀u∈Uθ(RB(u,x),PMA+B¯(X)(u)) =⋀u∈Uθ(RA(u,x),⋁v∈UT(RA(v,u),X(v))     ∨⋁v∈UT(RB(v,u),X(v)))  ∧⋀u∈Uθ(RB(u,x),⋁v∈UT(RA(v,u),X(v))      ∨⋁v∈UT(RB(v,u),X(v))) ≥⋀u∈Uθ(RA(u,x),⋁v∈UT(RA(v,u),X(v)))  ∧⋀u∈Uθ(RB(u,x),⋁v∈UT(RB(v,u),X(v))) =⋀u∈U ⋁v∈Uθ(RA(u,x),T(RA(v,u),X(v)))  ∧⋀u∈U ⋁v∈Uθ(RB(u,x),T(RB(v,u),X(v))) ≥⋁u∈U ⋀v∈Uθ(RA(u,x),      T(T(RA(v,x),RA(x,u)),X(v)))  ∧⋁u∈U ⋀v∈Uθ(RB(u,x),       T(T(RB(v,x),RB(x,u)),X(v))) ≥⋁v∈UT(RA(v,x),X(v))∧⋁v∈UT(RB(v,x),X(v)) =OMA+B¯(X)(x).
(5) According to item (5) in Proposition 7 and item (2) in Proposition 13, we can obtain
  (54) θ(PMA+B¯(X),OMA+B_(Y))=θ(OMA+B_PMA+B¯(X),Y)=θ(PMA+B¯(X),Y).
(6) This property can be proved by item (5).
(7) According to item (8) in Proposition 5 and item (2) in Proposition 13, we can have
  (55) OMA+B_Θ(a__,PMA+B¯(X))=Θ(a__,OMA+B_PMA+B¯(X))=Θ(a__,PMA+B¯(X)).

According to the properties, we can get the relation as follows:

(56) PM_(X)⊆PM¯(PM_(X))⊆OM_(X)⊆X⊆OM¯(X)⊆PM_(PM¯(X))⊆PM¯(X).

**Proposition 14 .**

Let (*U*, *R*_*A*_1__, *R*_*A*_2__,…, *R*_*A*_*n*__) be *T*-fuzzy approximation space; let *R*_*A*_*i*__  (*i* ∈ {1,2, 3,…, *n*}) be the different *T* fuzzy similarity relations, for all *x*, *y* ∈ *U*, *a*, *b* ∈ *I*, and *X*, *Y* ∈ *F*(*U*). Then, consider the following:

1. $\underline{OM_{\sum_{i=1}^{n}A_{i}}}(X)={⋃}_{i=1}^{n}\underline{R_{A_{i}}}\left(X\right)\;\;\bar{OM_{\sum_{i=1}^{n}A_{i}}}(X)={⋂}_{i=1}^{n}\bar{R_{A_{i}}}(X)$;
2. $\underline{PM_{\sum_{i=1}^{n}A_{i}}}(X)={⋂}_{i=1}^{n}\underline{R_{A_{i}}}(X)\;\;\bar{PM_{\sum_{i=1}^{n}A_{i}}}(X)={⋃}_{i=1}^{n}\bar{R_{A_{i}}}(X)$;
3. $\underline{PM_{\sum_{i=1}^{n}A_{i}}^{≽}}(X)\subseteq \underline{R_{A_{i}}^{≽}}(X)\subseteq \underline{OM_{\sum_{i=1}^{n}A_{i}}^{≽}}(X)$;
4. $\bar{PM_{\sum_{i=1}^{n}A_{i}}^{≽}}(X)⊇\bar{R_{A_{i}}^{≽}}(X)⊇\bar{OM_{\sum_{i=1}^{n}A_{i}}^{≽}}(X)$;

**Proof**

This proposition can be easily proved by Definitions 1, 3, and 8.

## 6. Case Study

Let us consider a fund investment issue. There are ten fund projects *x*_*i*_  (*i* = 1,2,…, 10) that can be considered. They can be evaluated from the view of profit factors. Profit factors can be classified into five factors, which are market, technology, management, environment, and production.  Table 1 is an evaluation information table about fund investment given by an expert, where *U* = {*x*_1_, *x*_2_, *x*_3_, *x*_4_, *x*_5_, *x*_6_, *x*_7_, *x*_8_, *x*_9_, *x*_10_}, *AT* = {Market, Technology, Management, Environment, Production}. For convenience, *a*_1_, *a*_2_, *a*_3_, *a*_4_, and *a*_5_ will stand for market, technology, management, environment, and production, respectively.

Now, we can use the following similarity functions to calculate the similarity relation between the objects *x*_*i*_, *x*_*j*_ as

(57) $$\begin{array}{c} R_{AT}\left(x_{i},x_{j}\right)=\{\begin{array}{cc} \overset{m}{\underset{k=1}{\sum }}\left(1-4\cdot \frac{\left|x_{i}^{a_{k}}-x_{j}^{a_{k}}\right|}{\left|\max a_{k}-\min a_{k}\right|}\right){\left(m\right)}^{-1}, & \\ \;\;\;\;\;\;\frac{\left|x_{i}^{a_{k}}-x_{j}^{a_{k}}\right|}{\left|\max a_{k}-\min a_{k}\right|}\leq 0.25; & \\ 0,\;\;\;\;\;\text{otherwise}. & \end{array} \end{array}$$

Let *A*_1_ = {Market, Technology} = {*a*_1_, *a*_2_}, *A*_2_ = {Management, Environment} = {*a*_3_, *a*_4_}, and *A*_3_ = {Production} = {*a*_5_}. So we can get three different *T*-fuzzy similarity relations as follows:

(58) RA1(xi,xj) ={((1−4·|xia1−xja1||max⁡⁡a1−min⁡⁡a1|) +(1−4·|xia2−xja2||max⁡⁡a2−min⁡⁡a2|)) × (2)−1, |xiak−xjak||max⁡⁡ak−min⁡⁡ak|≤0.25 (k=1,2);  0,     otherwise,RA2(xi,xj) ={((1−4·|xia3−xja3||max⁡⁡a3−min⁡⁡a3|) +(1−4·|xia4−xja4||max⁡⁡a4−min⁡⁡a4|)) ×(2)−1, |xiak−xjak||max⁡⁡ak−min⁡⁡ak|≤0.25 (k=3,4);  0,     otherwise,RA3(xi,xj) ={(1−4·|xia5−xja5|)|max⁡⁡a5−min⁡⁡a5|,|xia5−xja5||max⁡⁡a5−min⁡⁡a5|≤0.25; 0,otherwise.

From Table 1, we can get the *T*-fuzzy similarity matrices as follows:

(59) $$\begin{array}{c} R_{A_{1}}=\left[\begin{array}{cccccccccc} 1 & 0 & 0 & 0 & 0.674 & 0 & 0 & 0 & 0 & 0\\ 0 & 1 & 0 & 0 & 0 & 0 & 0 & 0 & 0 & 0\\ 0 & 0 & 1 & 0.727 & 0 & 0 & 0 & 0 & 0 & 0\\ 0 & 0 & 0.727 & 1 & 0 & 0 & 0 & 0 & 0 & 0\\ 0.674 & 0 & 0 & 0 & 1 & 0 & 0 & 0 & 0 & 0\\ 0 & 0 & 0 & 0 & 0 & 1 & 0 & 0 & 0 & 0\\ 0 & 0 & 0 & 0 & 0 & 0 & 1 & 0 & 0 & 0\\ 0 & 0 & 0 & 0 & 0 & 0 & 0 & 1 & 0 & 0.778\\ 0 & 0 & 0 & 0 & 0 & 0 & 0 & 0 & 1 & 0\\ 0 & 0 & 0 & 0 & 0 & 0 & 0 & 0.778 & 0 & 1 \end{array}\right],\\ R_{A_{2}}=\left[\begin{array}{cccccccccc} 1 & 0 & 0 & 0.133 & 0 & 0 & 0 & 0 & 0 & 0\\ 0 & 1 & 0.471 & 0 & 0 & 0 & 0 & 0.479 & 0 & 0\\ 0 & 0.471 & 1 & 0.547 & 0.471 & 0 & 0 & 0 & 0 & 0\\ 0.133 & 0 & 0.547 & 1 & 0.479 & 0 & 0 & 0.094 & 0 & 0\\ 0 & 0 & 0.471 & 0.479 & 1 & 0 & 0 & 0 & 0 & 0\\ 0 & 0 & 0 & 0 & 0 & 1 & 0 & 0 & 0 & 0\\ 0 & 0 & 0 & 0 & 0 & 0 & 1 & 0 & 0 & 0\\ 0 & 0.479 & 0 & 0.094 & 0 & 0 & 0 & 1 & 0 & 0\\ 0 & 0 & 0 & 0 & 0 & 0 & 0 & 0 & 1 & 0.667\\ 0 & 0 & 0 & 0 & 0 & 0 & 0 & 0.667 & 0 & 1 \end{array}\right],\\ R_{A_{3}}=\left[\begin{array}{cccccccccc} 1 & 0 & 0 & 0.077 & 0.282 & 0 & 0 & 0.897 & 0.179 & 0\\ 0 & 1 & 0.385 & 0 & 0 & 0.179 & 0 & 0 & 0 & 0.795\\ 0 & 0.385 & 1 & 0.487 & 0 & 0.795 & 0 & 0 & 0 & 0.590\\ 0.077 & 0 & 0.487 & 1 & 0 & 0.692 & 0 & 0.179 & 0 & 0.077\\ 0.282 & 0 & 0 & 0 & 1 & 0 & 0 & 0.179 & 0.897 & 0\\ 0 & 0179 & 0.795 & 0.692 & 0 & 1 & 0 & 0 & 0 & 0.385\\ 0 & 0 & 0 & 0 & 0 & 0 & 1 & 0 & 0 & 0\\ 0.897 & 0 & 0 & 0.179 & 0.179 & 0 & 0 & 1 & 0.077 & 0\\ 0.179 & 0 & 0 & 0 & 0.897 & 0 & 0 & 0.077 & 1 & 0\\ 0 & 0.795 & 0.590 & 0.077 & 0 & 0.385 & 0 & 0 & 0 & 1 \end{array}\right]. \end{array}$$

Taking *T*(*x*, *y*) = min⁡(*x*, *y*), the residual implication of *T* is

(60) $$\begin{array}{c} \theta \left(x,y\right)=\{\begin{array}{cc} 1, & x\leq y;\\ y, & x>y. \end{array} \end{array}$$

Assume that the comprehensive evaluation of a customer for these fund projects is a fuzzy set *X* = (0.5,0.6,0.3,0.8,0.5,0.2,0.4,0.7,0.2,0, 3). Then, the *T*-fuzzy lower and upper approximations of *X* are

(61) R_(A1)(X) =(0.5,0.6,0.3,0.3,0.5,0.2,0.4,0.3,0.2,0.3),R¯(A1)(X) =(0.5,0.6,0.727,0.8,0.5,0.2,0.4,0.7,0.2,0.7),R_(A2)(X) =(0.5,0.6,0.3,0.3,0.3,0.2,0.4,0.3,0.2,0.3),R¯(A2)(X) =(0.5,0.6,0.547,0.8,0.5,0.2,0.4,0.7,0.2,0.667),R_(A2)(X) =(0.5,0.3,0.2,0.2,0.2,0.2,0.4,0.5,0.2,0.2),R¯(A2)(X) =(0.7,0.6,0.487,0.8,0.5,0.692,0.4,0.7,0.5,0.6).  

Furthermore, we can get the optimistic and pessimistic multigranulation *T*-fuzzy lower and upper approximations of *X*, respectively,

(62) OMA1+A2+A3_(X) =(0.5,0.6,0.3,0.3,0.5,0.2,0.4,0.5,0.2,0.3),OMA1+A2+A3¯(X) =(0.5,0.6,0.487,0.8,0.5,0.2,0.4,0.7,0.2,0.6),PMA1+A2+A3_(X) =(0.5,0.3,0.2,0.2,0.2,0.2,0.4,0.3,0.2,0.2),PMA1+A2+A3¯(X) =(0.7,0.6,0.727,0.8,0.5,0.692,0.4,0.7,0.5,0.7).

From the above three granulations *A*_1_, *A*_2_, and *A*_3_, the projects must support optimistically the customer's comprehensive evaluation based on the degrees (0.5,0.6,0.3,0.3,0.5,0.2,0.4,0.5,0.2,0.3) and may support optimistically the customer's comprehensive evaluation based on the degrees (0.5,0.6,0.487,0.8,0.5,0.2,0.4,0.7,0.2,0.6); the projects must support pessimistically the customer's comprehensive evaluation based on the degrees (0.5,0.3,0.2,0.2,0.2,0.2,0.4,0.3,0.2,0.2) and may support optimistically the customer's comprehensive evaluation based on the degrees (0.7,0.6,0.727,0.8,0.5,0.692,0.4,0.7,0.5,0.7).

## 7. Conclusions

In this paper, we mainly presented the pessimistic multigranulation rough set model from the pessimistic multigranulation perspective by using *T*-fuzzy similarity relations in terms of triangular norms and studied the properties of optimistic and pessimistic multigranulation *T*-fuzzy lower and upper approximation operators. In the *T*-fuzzy approximation space (*U*, *R*_*A*_1__, *R*_*A*_2__,…, *R*_*A*_*n*__), the definitions of optimistic and pessimistic multigranulation *T*-fuzzy lower and upper approximation operators were recalled and proposed, respectively. It was obvious that the *T*-fuzzy lower and upper approximation operators which are defined on (*U*, *R*) were special cases of those of the two types of models. Furthermore, many interesting properties of the optimistic and the pessimistic multigranulation *T*-fuzzy rough sets models with respect to triangular norm have been explored. What is more, we researched the relationships among these approximation operators. The constructions of two new types of multigranulation rough set models over *T*-fuzzy similarity relations were meaningful in terms of the generalization of rough set theory. Finally, the models were illustrated by a case study about the evaluation of fund projects.

## Figures and Tables

**Table 1:** An information system about fund investment.

*U*	Market	Technology	Management	Environment	Production
*x*_1_	73	88	75	85	74
*x*_2_	86	84	79	60	54
*x*_3_	84	71	81	68	60
*x*_4_	87	69	79	74	65
*x*_5_	68	87	83	76	81
*x*_6_	71	62	80	91	62
*x*_7_	92	52	75	39	43
*x*_8_	55	72	75	62	73
*x*_9_	60	55	65	72	82
*x*_10_	55	68	72	62	56
